# 750 Discharge to Door Metrics: Collaboration Matters!

**DOI:** 10.1093/jbcr/irae036.292

**Published:** 2024-04-17

**Authors:** Jenny Granger, Nora Bujanda-Sotelo, Tiffany Hockenberry, Karen J Richey, Kevin N Foster

**Affiliations:** Valleywise Health, Phoenix, AZ; Valleywise Health Medical Center, Goodyear, AZ; Arizona Burn Center Valleywise Health, Phoenix, AZ; Valleywise Health, Phoenix, AZ; Valleywise Health Medical Center, Goodyear, AZ; Arizona Burn Center Valleywise Health, Phoenix, AZ; Valleywise Health, Phoenix, AZ; Valleywise Health Medical Center, Goodyear, AZ; Arizona Burn Center Valleywise Health, Phoenix, AZ; Valleywise Health, Phoenix, AZ; Valleywise Health Medical Center, Goodyear, AZ; Arizona Burn Center Valleywise Health, Phoenix, AZ; Valleywise Health, Phoenix, AZ; Valleywise Health Medical Center, Goodyear, AZ; Arizona Burn Center Valleywise Health, Phoenix, AZ

## Abstract

**Introduction:**

Timely hospital discharges are an essential part of patient satisfaction and emergency department throughput. The discharge process for burn patients is complex and delays can be both frustrating and anxiety producing. In addition, delayed discharges affect the financial bottom line, patients that are discharged late in the day still require care but if not on unit when midnight strikes, hospitals are unable to charge for that inpatient day. Our institutional benchmark is ≤ 90 minutes from time of written discharge order to patient leaving the center. The purpose of this quality improvement (QI) project was to evaluate our center’s current status, identify process barriers, develop interventions, and decrease delayed discharges (DC).

**Methods:**

This was a retrospective chart review examining the relationship between placement of the DC order and the following elements, terminal dressing change, rehabilitation clearance, pending care management/transportation needs, and discharge destination.

**Results:**

A total of 96 charts were reviewed and 47% (n=45) of center DC exceeded 90 minutes, with an average time to DC of 3 hours 33 minutes. Of the 45 patients with delayed DC, only 17 (38%) had all elements of the DC process completed prior to the order being placed. Those who did not have all elements completed were split evenly between having 1-2 tasks pending. The most frequently uncompleted tasks were pending care management/transportation needs (47%), completion of terminal dressing change (33%) and rehabilitation clearance (13%). Patients with timely DC were more likely to have been DC home (78%) compared to untimely DC (62%). A multidisciplinary task force was formed, and the following interventions were initiated; formalization of daily Clinical Resource Leaders report to include estimated discharge date, discharge destination, status of family/support discharge teaching and targeted education for the ordering clinicians, nurses, rehabilitation and the care management team. Within 4 months of instituting these interventions, delayed DC dropped to 25% and at 6 months were down to 15%. These results have been sustained and over the past 10 months less than 10% of burn DC have exceeded the 90-minute benchmark.

**Conclusions:**

Overwhelmingly, this QI project demonstrated the importance of a fully integrated, collaborative approach to timely discharges. Discharge planning begins on admission and is an ongoing process. Establishing an estimated date of discharge, ensuring teaching is done prior to the day of discharge, ensuring wound care is complete as well as all rehabilitation and care management needs are met, are essential to making sure the patient is ready for a safe and timely discharge.

**Applicability of Research to Practice:**

Discharge collaboration increases patient satisfaction, decreases discharge times, improves hospital throughput, and improves the financial health of the organization.

